# Cross-linguistic phonological activation in Chinese– English–Korean trilinguals

**DOI:** 10.3389/fpsyg.2026.1894710

**Published:** 2026-09-22

**Authors:** Jung Hyun Lim, Hee-Don Ahn

**Affiliations:** 1 Institute of Multilingualism and Multiculturalism, Konkuk University, Seoul, Republic of Korea; 2 Department of English Language and Literature, Konkuk University, Seoul, Republic of Korea

**Keywords:** different-script languages, homophones, non-selective activation, phonological priming, proficiency, trilingual lexical processing

## Abstract

This study investigates whether phonological similarity alone can drive cross-linguistic activation across non-native languages in trilingual lexical processing. Using a masked priming lexical decision task, we examined phonological priming from English (L2) to Korean (L3) in Chinese–English–Korean unbalanced trilinguals. Critically, homophone pairs were employed to minimize semantic and orthographic overlap, allowing for a clearer examination of phonological priming than previous studies relying on cognates. The results showed a robust phonological priming effect between non-native languages, suggesting that phonological information alone can trigger cross-linguistic activation. In addition, proficiency was treated as a continuous variable, with L2 and L3 proficiency showing dissociable effects: higher L3 proficiency facilitated lexical access, whereas higher L2 proficiency was associated with slower response times, suggesting increased cross-language competition. Overall, the present study demonstrates that phonological representations are shared between non-native languages, supporting non-selective cross-linguistic activation in trilingual lexical processing, and highlights the important role of proficiency in lexical processing. These findings are discussed within the framework of bilingual and trilingual models of lexical access and provide further insights into the development of more refined models of trilingual lexical processing.

## 1 Introduction

Over the past several decades, a considerable amount of research on bilingual lexical processing has examined how two languages are represented and processed in the bilingual mind. This line of research has consistently shown that both languages are activated in parallel in a non-selective manner even when only the target language is relevant for the response (Dimitropoulou et al., 2011; Duyck et al., 2004; Gollan et al., 1997; Jiang and Forster, 2001; Nakayama et al., 2016; Van Hell and de Groot, 1998). To account for these phenomena, several influential models have been proposed, including the Bilingual Interactive Activation Plus (BIA+) model (Dijkstra and van Heuven, 2002), the Revised Hierarchical Model (RHM) (Kroll and Stewart, 1994), and the Multilink model (Dijkstra et al., 2019), which explain how languages are organized and represented across two lexicons. Recently, building on extensive research in bilingual processing, there has been increasing research on trilingual language processing, particularly with regard to how multiple languages interact during lexical access. Since trilingual processing entails more complex interactions among three linguistic systems than bilinguals, it often raises challenges for experimental design and the control of variables. Drawing on insights from bilingual research, however, researchers have begun to extend this line of inquiry to trilinguals, aiming to understand how activation emerges across three languages and to identify the factors that influence this process.

Much of the existing research on trilingual lexical processing has typically focused on cognates and noncognates to investigate how trilingual speakers process and represent three languages (Aparicio and Lavaur, 2016; Ellias et al., 2025; Lemhöfer et al., 2004; Li et al., 2024; Song et al., 2025; Yang et al., 2025; Yu and Kong, 2026). In particular, cognates have been widely used as a diagnostic tool to examine whether non-target languages are activated when participants perform a task in one language, as they share semantic, phonological, and often orthographic features across languages. Findings from such studies have generally reported cognate facilitation effects, although the magnitude and robustness of these effects can vary depending on factors such as language direction and typological distance (Lemhöfer et al., 2004; Li et al., 2024; Song et al., 2025; Yu and Kong, 2026). These findings suggest that lexical representations across multiple languages can be co-activated across both same-script and different-script languages, consistent with observations from bilingual processing research. However, because cognates involve overlaps at multiple representational levels simultaneously, it is often difficult to determine which level of representation, i.e., phonological, orthographic, or semantic, is primarily responsible for the observed cross-linguistic activation. As such, the specific locus of this activation is not yet fully understood in trilingual processing.

In contrast, this issue has been examined in bilingual lexical processing. A line of research in bilingual lexical processing explored whether phonological information in one language activates lexical access in the other language, by employing various types of tasks, such as a lexical decision task, a naming task, or a sentence reading task. Although results showed that phonological effects can be dependent on task type or language direction, this line of research suggests that phonological representations are shared between languages, and phonological access is automatic and occurs early in word recognition (Jiang, 2019; Kim and Davis, 2003; Lim and Christianson, 2023; Nakayama et al., 2012, 2014; Zhang et al., 2023; Zhou et al., 2010). While there are many studies that investigated phonological priming effects in bilingual word recognition, it remains unclear whether the effects extend to trilingual speakers, especially when the languages involve different scripts (cf. Fridman and Meir, 2026; Lim and Ahn, 2024). Addressing this gap is important, as phonological priming offers a more direct means of testing cross-linguistic interaction at the form level, independent of semantic overlap.

The present study addresses this issue by examining cross-linguistic phonological activation in Chinese–English–Korean unbalanced trilingual speakers. Specifically, we investigate whether phonological overlap between non-native languages influences lexical decision in L3, and whether such effects are modulated by language proficiency. By focusing on phonological similarity in the absence of semantic relatedness, the study aims to provide a clearer understanding of cross-linguistic activation mechanisms in trilingual lexical processing. In addition, previous bilingual and multilingual studies have repeatedly shown that proficiency plays a crucial role in modulating cross-language activation (e.g., Duyck et al., 2004; Lim and Christianson, 2023; Lim and Ahn, 2024). Yet, very few studies have examined L2 and L3 proficiency as continuous factors influencing trilingual lexical processing.

## 2 Theoretical background

Cross-linguistic activation has been extensively reported in bilingual lexical processing, providing strong evidence for nonselective language access within frameworks such as the BIA+ model (Dijkstra and van Heuven, 2002). In contrast, the mechanisms underlying lexical organization and representation in trilinguals remain relatively underexplored when compared to the well-established literature on bilingual processing, despite a growing body of recent research in this area. Recently, an increasing number of studies have begun to examine how languages are represented and co-activated in trilingual speakers, extending findings from bilingual research (Aparicio and Lavaur, 2016; Ellias et al., 2025; Lemhöfer et al., 2004; Li et al., 2024; Song et al., 2025; Yang et al., 2025; Yu and Kong, 2026; Xue et al., 2022). These studies suggest that lexical access in trilingual speakers may involve more complex interactions among three linguistic systems, raising the possibility that activation patterns differ from those observed in bilingual contexts.

A growing body of research on trilingual lexical processing has focused on cross-linguistic activation through cognate words which share forms (phonology or orthography) and meaning across languages. For example, one of the early studies, Lemhöfer et al. (2004) investigated cognate facilitation in trilingual word recognition using a lexical decision task with Dutch–English–German speakers. Their study systematically compared three types of stimuli: non-cognates, double cognates that overlapped across two languages, and triple cognates that shared form and meaning across all three languages. The results found additive cognate facilitation effects, such that double cognates were recognized faster than non-cognates, and critically, triple cognates were processed even more rapidly than double cognates. This cumulative facilitation effect suggests that lexical activation is not restricted to a single non-target language, but can extend across multiple languages simultaneously, providing strong support for nonselective lexical access in trilinguals. However, because cognates inherently involve shared semantic, phonological, and orthographic representations, and because the languages used in the study are written in the same script, it remains challenging to disentangle the relative influences of different linguistic levels in cross-linguistic activation.

Another recent study by Li et al. (2024) investigated masked translation priming effects in Chinese–English–Japanese trilinguals using triple different-script cognates in a lexical decision task, examining multiple priming directions including L1–L2, L1–L3, and L2–L3. Their results showed that priming effects were observed only when primes originated from a stronger language, such as L1–L2, L1–L3, and L2–L3. Notably, Experiment 3 is related to the current study in a way that it directly tested L2-to-L3 priming, providing evidence that L2 primes strongly facilitated the lexical decision in L3. Given that the priming effects were obtained using cognate translation equivalents, the observed facilitation likely reflected the combined influence of semantic and phonological overlap, rather than isolating phonological similarity *per se*. Similarly, Ellias et al. (2025) investigated cognate facilitation in Arabic-Hebrew-English trilinguals with double, triple cognates, just as Lemhöfer et al. (2004), and further examined whether such effects are modulated by task demands using different tasks, including a semantic relatedness task and a lexical decision task. The results showed that cognate facilitation was more likely to emerge in tasks that required deeper semantic processing, whereas in tasks (i.e., lexical decision task) that placed less emphasis on meaning, the effect was weaker or absent. The study provided novel insights into cross-linguistic activation in trilinguals, showing that it is sensitive to task demands and the types of information (semantic, phonological, orthographic). Although this work yields strong evidence that cognates can lead to the co-activation of multiple languages, even across languages with different scripts, the primary mechanism examined was still cognate-based overlap, where semantic and form similarity are intertwined. As a result, the study leaves open the question of whether phonological representations alone can independently drive cross-linguistic activation in trilinguals.

A second line of research has examined semantic priming and interactions between languages in trilingual lexical processing, particularly with respect to how previously acquired languages (L1 and L2) affect L3 word recognition. Song et al. (2025), for instance, examined whether the recognition of L3 words was influenced by previously acquired languages with Chinese–English–Spanish trilinguals, using cognates, orthographic neighbors, translations, and unrelated words. The study specifically tested the applicability of the Parasitic Model (Hall, 2002) of vocabulary acquisition that assumes that L3 lexical representations initially rely on previously acquired languages (L1 and/or L2), rather than being independently represented. Findings showed robust semantic priming from L1 but more limited effects from L2, suggesting that L3 lexical representations may be more strongly anchored to L1 conceptual systems, probably because L1 is the more established language. At the same time, the study also found that form overlap, particularly orthographic similarity between L2 English and L3 Spanish, could influence L3 word recognition, indicating that L2 may still contribute to L3 processing when there is sufficient surface similarity. This study is relevant to the present research because it directly addresses L2–L3 interaction in a masked priming lexical decision task, just as Li et al. (2024). And yet again, its focus remains largely on semantic and orthographic relations with relatively little attention paid to phonological activation.

In a similar vein, Yu and Kong (2026) demonstrated that semantic activation in trilinguals is directionally asymmetric, with priming effects emerging only in specific language directions. They employed a masked translation priming with an animacy judgment task, focusing on semantic processing in Portuguese-English-Chinese trilingual speakers. Their findings highlight that cross-linguistic activation is not consistent across languages, but depends on factors such as language status, proficiency, instructional experience, and typological distance. In particular, the L2–L3 effect suggests that L2 English can serve as a mediating language for L3 Chinese, especially when English has higher proficiency and functions as an instructional or bridge language. Their findings therefore support the view that L2 may play a privileged role in trilingual lexical processing, consistent with the L2 Status Factor hypothesis (Bardel and Falk, 2007), which proposes that L3 representations are preferentially linked to L2 rather than L1. The study also suggest that typological distance may constrain direct L1–L3 interactions, as reflected in the absence of a reliable L1–L3 priming effect. While Yu and Kong (2026) focused on semantic translation priming, the present study examines whether L2 phonological information can influence L3 visual word recognition, thereby extending this line of work to phonological activation in trilingual lexical processing.

More direct evidence regarding phonological activation in trilinguals is still scarce compared to the studies focusing on cognate facilitation and semantic activation. One of the few studies to examine phonological activation is Lim and Ahn (2024), who investigated cross-linguistic phonological priming in Chinese–English–Korean unbalanced trilinguals using a masked-priming lexical decision task. Specifically, they examined whether phonological information in L3 could influence word recognition in L2, thereby testing the possibility of backward cross-linguistic influence. Their findings revealed a marginal trend toward phonological priming (*p* = 0.08) and marginally reliable interactions with language proficiency were observed, tentatively indicating that higher proficiency in L2 and L3 may strengthen cross-linguistic phonological activation. The authors noted that this weak priming effect could be interpreted in light of the Parasitic Model (Hall, 2002), whereby L3 representations, being less firmly established, may not yet exert strong influence on L2 processing, in line with previous findings showing little to no priming effects when activation originates from a less dominant language. Despite the effects being marginal, the study is particularly important in that it isolates phonological similarity from semantic overlap, pointing to the possibility that phonological representations alone may contribute to cross-linguistic activation in trilingual lexical processing. However, since the study showed only a marginal trend toward phonological priming, further investigation is needed to establish the robustness of these findings and to determine whether the observed trend is associated with the directional asymmetry reported in previous trilingual lexical research (Li et al., 2024; Yu and Kong, 2026).

Recently, Fridman and Meir (2026) employed an eye-tracking visual world paradigm to investigate cross-linguistic phonological co-activation in trilingual speakers. Using Russian–Hebrew–English trilinguals, they examined whether auditory input in one language could activate phonologically similar competitors in the other two languages. Their findings showed that cross-linguistic phonological competitors influenced processing, as reflected in increased fixation to competitor items and delayed reaction times, particularly in weaker-language contexts, suggesting that multiple languages can be co-activated during spoken word recognition. These findings were obtained using an online eye-tracking paradigm with auditory input, and this raises the question of whether similar patterns can be observed in masked priming lexical decision tasks with visual input.

Taken all together, recent trilingual processing research has made important progress in explaining how multiple languages interact during lexical processing and learning. They suggest that cross-linguistic activation in trilinguals is somewhat robust, broadly consistent with patterns observed in bilingual processing. At the same time, it appears to be governed by a more complex interplay of factors, including language status, typological distance, and language experience. However, most studies have focused on semantic and orthographic overlap, often through cognates or translation equivalents. Even in studies that consider phonological similarity, phonological effects are often embedded within broader cognate effects or form similarity, making it difficult to isolate the contribution of phonological representations themselves. This leaves an important gap in the literature. Yet relatively few studies have directly tested whether L2 phonological representations can influence L3 word recognition in the absence of semantic overlap, particularly in different-script language combinations. Moreover, the role of proficiency in modulating phonological activation has not been systematically investigated in the trilingual literature.

To address this gap, the present study examines phonological priming from English (L2) to Korean (L3) in Chinese–English–Korean trilinguals, thereby complementing Lim and Ahn (2024), which investigated the reverse direction. Using phonologically related but semantically unrelated prime–target homophone pairs, the study isolates phonological activation more directly than previous cognate-based research. The study also considers both L2 and L3 proficiency to investigate how language proficiency modulates phonological processing in trilingual lexical access. Furthermore, building on Lim and Ahn (2024), the present study provides a basis for a broader discussion of phonological activation across languages and offers further insight into how such effects vary as a function of language direction and proficiency in trilingual lexical processing.

### 2.1 The present study

Previous research on multilingual lexical processing has primarily focused on cognate effects and semantic co-activation across languages. Although cross-language phonological priming has been extensively examined in bilinguals as mentioned above, relatively little is known about whether such phonological activation extends to trilingual lexical processing, particularly across different-script languages. To fill this gap in the trilingual lexical processing research, the present study investigated whether English (L2) phonological information influences Korean (L3) word recognition by native Chinese speakers. Specifically, we address the following questions:

1. Is there a reliable phonological priming effect from English (L2) to Korean (L3)?
2. How do L2 and L3 proficiency modulate phonological activation in L3 word recognition?
3. Does language proficiency interact with the priming effect, indicating differential sensitivity to cross-language phonological activation?

Based on previous findings in bilingual and trilingual lexical processing (Kim and Davis, 2003; Li et al., 2024; Lim and Christianson, 2023; Song et al., 2025; Zhang et al., 2023; Zhou et al., 2010), we hypothesized that English (L2) homophone primes would facilitate Korean (L3) word recognition, such that responses would be faster in the related condition than in the unrelated condition, reflecting non-selective phonological activation across different-script languages. We also predicted that language proficiency would have influences on either the speed of word recognition or the magnitude of priming effects, reflecting processing dynamics in L3 word recognition. It is predicted that L2 proficiency would modulate L3 word recognition, as higher proficiency is typically linked to more robust and automatic activation in lexical processing. We further predicted that higher L3 proficiency would be associated with faster overall response times, reflecting more efficient lexical access in the target language. This prediction is consistent with previous research reporting that increased language proficiency usually enhances processing efficiency and leads to faster and more automatic visual word recognition (e.g., Freeman and Marian, 2022; Hopp, 2010; Mitsugi, 2018; see also Dijkstra and van Heuven, 2002).

The present study was designed as a parallel investigation to Lim and Ahn (2024). It closely matched the previous study in participant pool, proficiency profiles, experimental design, and stimuli, with the only difference being the prime–target language direction. This design enables a direct comparison of priming effects across language directions and provides a basis for examining how phonological activation operates in unbalanced trilingual speakers, as well as how it varies as a function of language direction and proficiency.

## 3 Methods

### 3.1 Participants

Forty-two participants voluntarily took part in a masked-priming lexical decision task and received monetary compensation for their participation. The study was approved by the Institutional Review Board of Konkuk University, and all procedures were conducted in accordance with relevant ethical guidelines. They reported normal or corrected-to-normal vision and provided informed consent prior to participation. All participants were native speakers of Chinese and were either undergraduate or graduate students enrolled at a university in Seoul, Korea (mean age = 24 years). Participants had learned English as a second language (L2) through private or formal education around the age of 9–10 and began learning Korean (L3) at around age 20, when they entered university in Korea. English proficiency was assessed using a cloze test based on a short narrative passage, in which a word was omitted every 6–7 words and participants selected the appropriate word to fill each blank, with a maximum score of 40 [adapted from Lim and Christianson (2013)]. Korean proficiency was assessed using a mini version of the Test of Proficiency in Korean (TOPIK), adapted from official sample items available on the TOPIK website (Ahn and Jang, 2019; Lim and Ahn, 2024). The mean English proficiency score was 22.60 (SD = 6.50; maximum score = 40), and the mean Korean proficiency score was 15.27 (SD = 3.23; maximum score = 20). Both measures were used as objective, performance-based indices of English and Korean proficiency. The resulting proficiency scores were used as continuous predictors in the statistical analyses.

### 3.2 Materials and design

The experimental items were adapted from those used in Lim and Ahn (2024). Critical stimuli consisted of 30 Korean target words, each preceded by either phonologically related English primes or unrelated English primes. All target words were concrete nouns, selected to ensure semantic transparency and to minimize variability in lexical processing. The stimulus pairs were selected to maximize phonological overlap across English and Korean while avoiding semantic relatedness. For example, the English word “*bee*”/*bi:/* and the Korean word “비”/*pi/* (meaning “rain”) share highly similar phonological forms, including comparable onset–rime structure, but are semantically unrelated. To minimize potential confounding effects of lexical frequency and length, primes and targets were carefully matched across conditions. All Korean target words were monosyllabic, and their word frequencies were obtained from a Sejong National Corpus–based database (Korea University), with a mean log frequency of 3.24. The English primes were 3.5 letters long on average, and their frequencies were obtained from SUBTLEX-US, with a mean log frequency of 3.52. Unrelated English primes were controlled for frequency (mean log frequency = 3.59) and syllable length as well. Although frequency measures were derived from language-specific corpora, the stimuli were selected to avoid substantial frequency imbalances across conditions. In addition to the homophone pairs, thirty filler word pairs that were neither phonologically nor semantically related were included; these fillers were also matched to the critical items on frequency and length. Sixty nonwords that conformed to Korean orthographic and phonotactic constraints were also included to ensure a balanced distribution of “yes” and “no” responses and to minimize participants’ awareness of the experimental purpose. Two counterbalanced lists of the experimental stimuli were created such that English homophone primes were paired with related Korean targets in List 1 and with unrelated targets in List 2, and vice versa. Participants were randomly assigned to one of the two lists, with 21 participants in each list. The full list of stimuli is provided in the Supplementary materials.

### 3.3 Procedure

The experiment was conducted individually in a quiet laboratory setting. Participants were seated in front of a computer equipped with E-Prime 3.0 software. Each trial began with a fixation cross (+) presented at the center of the screen for 300 ms, followed by a forward mask (#####) presented for 500 ms. Then, a lower-case English prime was presented briefly (50 ms), followed by a Korean target. The English prime was completely masked by the onset of the Korean target. Participants were then instructed to decide as quickly and accurately as possible whether the Korean target was a real Korean word or nonword by pressing one of two designated keys (e.g., “1” for Yes and “0” for No). No feedback was provided during the main experiment. The experiment consisted of 120 trials, preceded by six practice trials designed to familiarize participants with the task. Trial order was randomized for each participant. Both reaction times and response accuracy were recorded. After the main lexical decision task, participants completed an English cloze test and a Korean Mini TOPIK test, followed by a brief oral interview to confirm their familiarity with the experimental words. The whole procedure took approximately 35–45 min.

## 4 Results

Data from two participants were excluded: one due to low accuracy on the lexical decision task (below 70%), and the other because an insufficient number of valid trials remained after preprocessing, leaving a final sample of 40 participants. Table 1 presents mean response times (with standard deviations in parentheses) and error rates for the phonologically related and unrelated conditions. Descriptive statistics showed that mean latencies were shorter in the related condition (*M* = 792.91 ms) than in the unrelated condition (*M* = 851.80 ms), yielding a priming effect of 58.89 ms. Error rates were higher in the unrelated condition (12.2%) than in the related condition (9.5%). Accuracy was analyzed using a logistic mixed-effects model with random intercepts for subjects and items. Accuracy did not differ significantly between conditions (χ^2^ = 1.84, *p* = 0.18), indicating no reliable difference between the related and unrelated conditions. This suggests that the reaction time effect was not driven by a speed–accuracy trade-off.

**TABLE 1:** Mean reaction times (ms) and error rates (%) by prime condition.

Prime condition	Mean RT (ms)	Error rates (%)	Example
Related	792.91 (289.87)	9.5 (2.9)	bee - 비/pi/
Unrelated	851.80 (360.81)	12.2 (3.2)	son - 비/pi/
Priming effect (unrelated - related)	58.89		

To test the priming effect statistically, response latencies were analyzed using a linear mixed-effects model in R (Baayen et al., 2008). Response latency data were log-transformed and trimmed by excluding incorrect responses and outliers (e.g., values exceeding ±2.5 SD from the mean of each condition), resulting in the exclusion of approximately 4% of the data. The trimmed data were analyzed using a linear mixed-effects model with random intercepts for subjects and items. An initial full model included prime condition, Korean proficiency, English proficiency, and their interactions. Model comparisons using likelihood ratio tests indicated that the interaction terms did not significantly improve model fit (all ps > 0.05). Therefore, the interaction terms were removed, leaving prime condition, Korean proficiency, and English proficiency as fixed effects. As prime condition varied within subjects, we also evaluated a model including a by-subject random slope for prime condition. However, this model resulted in a singular fit, with the variance of the random slope estimated to be essentially zero. We therefore retained the random-effects structure with random intercepts for subjects and items in the final model. Table 2 presents the results of a linear mixed-effects model of the response times.

**TABLE 2:** Fixed effects of the linear mixed-effects model for response latencies in the lexical decision task.

Prime condition	Estimate	SE	df	*t*	*p*
(Intercept)	2.878	0.015	60.5	187.39	<0.001^***^
Prime condition (unrelated vs. related)	0.024	0.008	1038	3.16	0.002^**^
Korean proficiency	−0.009	0.004	38.9	−2.23	0.032*
English proficiency	0.006	0.002	37.6	3.35	0.002^**^

*p < 0.05,

^**^p < 0.01,

^***^p < 0.001.

The model revealed a significant effect of prime condition, with slower responses in the unrelated condition compared to the related condition (β = 0.024, SE = 0.008, *t* = 3.16, *p* = 0.002), reflecting the mean response times reported above. Korean proficiency also showed a significant effect, such that higher proficiency was associated with faster reaction times (β = −0.009, SE = 0.004, *t* = −2.23, *p* = 0.032). In contrast, English proficiency had a significant effect on reaction times, with higher proficiency linked to slower responses (β = 0.006, SE = 0.002, *t* = 3.35, *p* = 0.002). These results provide evidence for a L2–L3 phonological priming effect in trilingual lexical processing, with no evidence that the effect varied as a function of L2 or L3 proficiency. Interestingly, English (L2) and Korean (L3) proficiency exhibited significant effects in opposite directions, such that higher L3 proficiency associated with faster processing, whereas higher L2 proficiency unexpectedly led to slower responses, in contrast to the original prediction. Figures 1, 2 illustrate the predicted reaction times across levels of English and Korean proficiency, respectively. Together, these findings indicate that English and Korean proficiency were associated with response times in opposite directions.

**FIGURE 1:**
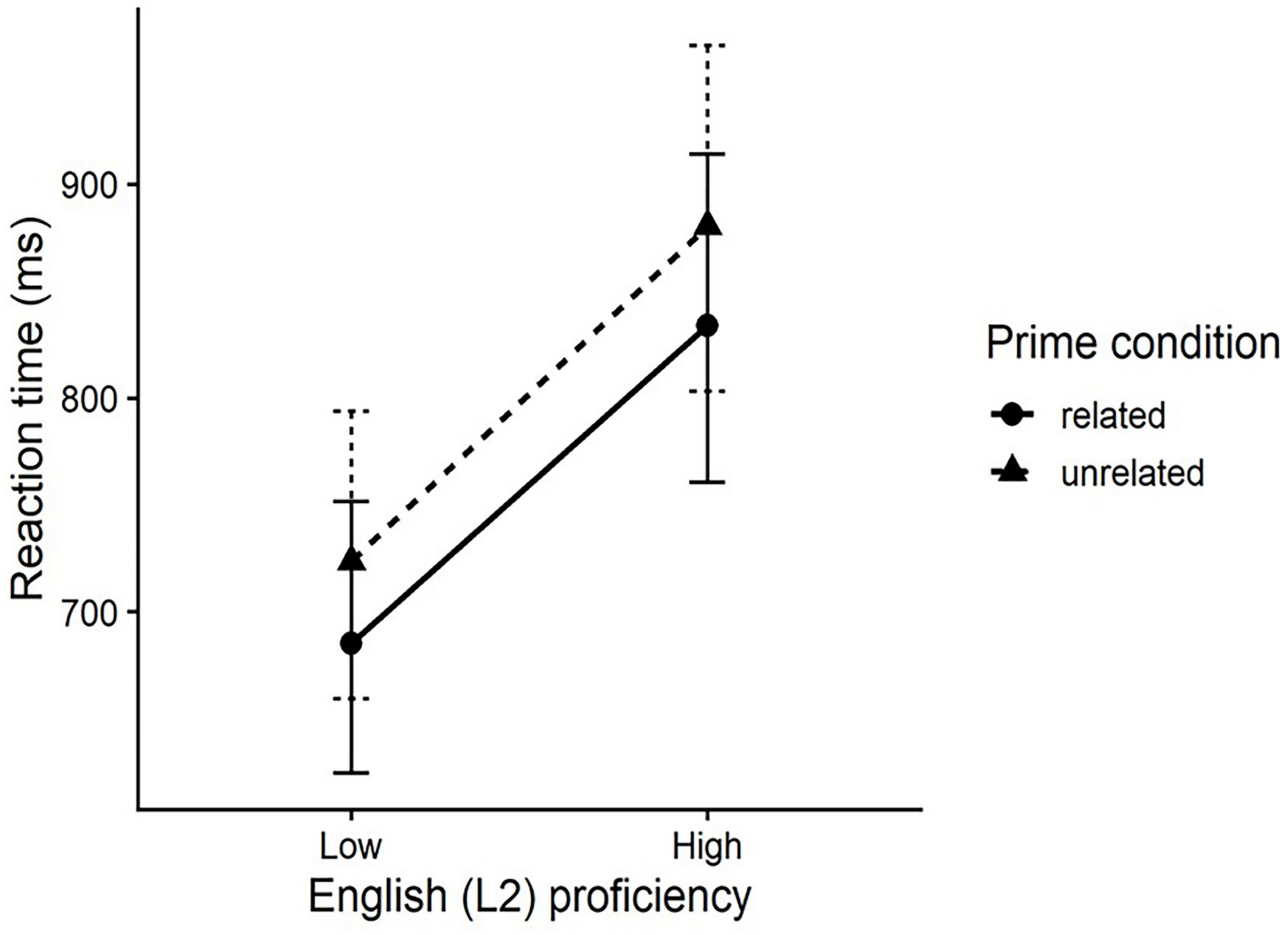
Predicted reaction times (ms) as a function of English (L2) proficiency. Error bars represent 95% confidence intervals.

**FIGURE 2:**
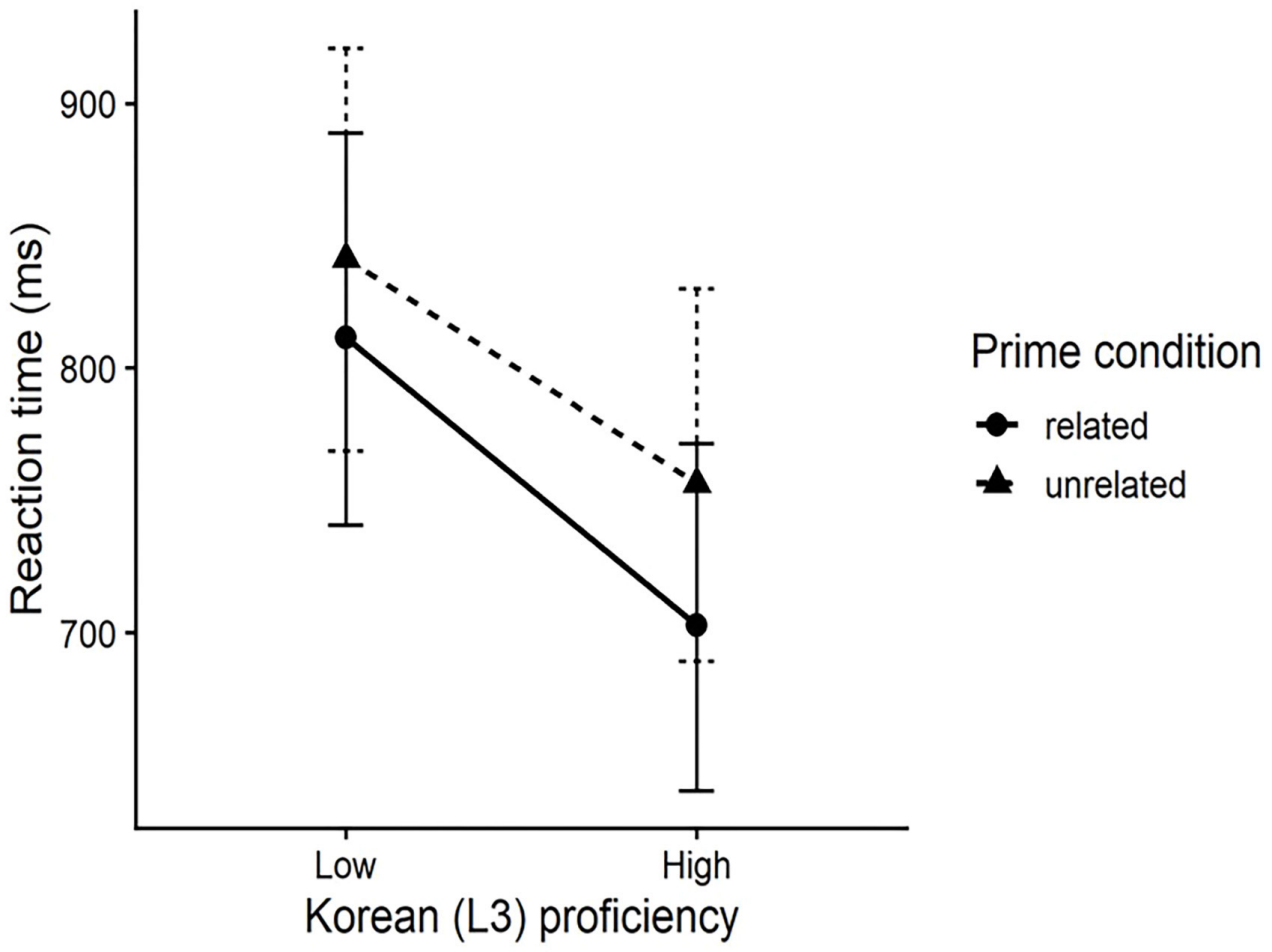
Predicted reaction times (ms) as a function of Korean (L3) proficiency. Error bars represent 95% confidence intervals.

## 5 Discussion

In the present study, we set out to investigate whether English (L2) phonological information influences Korean (L3) word recognition in Chinese-English-Korean unbalanced trilinguals using a masked-prime lexical decision task. Specifically, the study examined whether L2 primes that were phonologically related to L3 targets would facilitate L3 word recognition compared to unrelated primes, and whether such effects would be modulated by L2 and L3 proficiency. While much of the recent trilingual literature has examined cross-linguistic activation through cognates and translation equivalents, in which phonological overlap is sometimes discussed alongside semantic and orthographic similarity (Ellias et al., 2025; Li et al., 2024; Song et al., 2025; Xue et al., 2022; Yu and Kong, 2026), relatively less attention has been paid to the independent role of phonological activation across non-native languages in trilingual lexical processing. By isolating phonological overlap in the absence of semantic relatedness, the present study contributes to this line of research by extending discussions of non-selective lexical access beyond bilingual processing and providing a more direct examination of cross-linguistic interaction at the phonological level.

Three major findings emerged from the present experiment. First, a robust homophone priming effect was observed across non-native languages, indicating that phonological similarity alone can trigger cross-linguistic activation. Second, higher L2 proficiency was associated with increased processing costs in L3 lexical decision, suggesting that stronger representations in the non-target language may have increased competition during lexical processing. Third, higher L3 proficiency facilitated lexical processing, reflecting increased efficiency in processing the target language.

### 5.1 Phonological priming across non-native languages

First, the present study found a significant phonological priming effect from English (L2) to Korean (L3) in Chinese–English–Korean unbalanced trilingual speakers. The significant main effect of prime condition demonstrates that L2 homophone primes facilitate lexical decision in L3, providing evidence that phonological information alone can support cross-linguistic activation across non-native languages. These findings are consistent with the BIA+ model (Dijkstra and van Heuven, 2002), which assumes non-selective and interactive activation across linguistic levels and languages, and suggest that such non-selective phonological activation can also occur between L2 and L3 in trilingual speakers. The present findings are also consistent with previous bilingual studies suggesting shared phonological representations across languages (Jiang, 2019; Kim and Davis, 2003; Lim and Christianson, 2023; Nakayama et al., 2012; Zhang et al., 2023; Zhou et al., 2010). Given that phonological priming has remained relatively underexplored in trilingual lexical processing, the present study extends this line of research by providing evidence that phonological representations can also be activated between two non-native languages, L2 and L3, in trilingual speakers. In addition, the L2-to-L3 priming effect observed here is in line with some of the trilingual studies (Li et al., 2024; Song et al., 2025), who reported similar effects using cognate translation equivalents in a masked priming lexical decision task, while at the same time extending these findings by showing that phonological information alone is sufficient to influence lexical access in a third language.

The present study is particularly meaningful in relation to Lim and Ahn (2024), which motivated the current investigation. It complements and extends their findings, which reported relatively weak phonological priming from Korean (L3) to English (L2) in Chinese-dominant trilinguals. Specifically, Lim and Ahn (2024) reported only marginal phonological priming from L3 to L2 (21.67 ms difference, *p* = 0.08), whereas the present study observed a robust priming effect from L2 to L3 (58.89 ms difference, *p* = 0.002). The shift from marginal to significant priming suggests that the magnitude of phonological priming effects may vary as a function of language direction and the characteristics of the languages involved.

One possible explanation for this asymmetry comes from the Parasitic Model (Hall, 2002; Ecke, 2015). Within this framework, later-acquired languages develop parasitically on previously established linguistic systems. Given that all participants reported earlier exposure to English (L2) than to Korean (L3), English phonological representations may be more strongly entrenched and more readily accessible during lexical processing. Under this account, activation originating from English is likely to spread more efficiently across languages than activation originating from Korean. Although the present study does not directly test the Parasitic Model, the asymmetry between the present findings and those of Lim and Ahn (2024) is compatible with the idea that cross-linguistic activation may operate more effectively from earlier-acquired and more strongly established linguistic systems toward later-acquired and less established ones. Furthermore, these combined findings are in line with previous studies on semantic and cognate processing in trilinguals, which reported directional asymmetries in cross-linguistic activation depending on language status, learning history, and typological relationships (Fridman and Meir, 2026; Li et al., 2024; Yu and Kong, 2026). Taken together, these results extend this asymmetric pattern to phonological priming, suggesting that similar directional tendencies may also be present at the phonological level.

Another possible explanation for the observed asymmetry concerns not whether phonological representations are shared across languages, but how rapidly and sufficiently phonological information becomes available during early word recognition. Li et al. (2024) argued that phonological priming critically depends on the speed with which the phonology of both the prime and the target is activated. In their study, this issue was discussed mainly in relation to logographic scripts, where relatively weak orthography-to-phonology mappings may delay phonological activation. Although neither the present study nor Lim and Ahn (2024) involved logographic scripts, the same general processing principle may still be relevant. That is, phonological priming may be reduced whenever the prime provides limited or weak phonological cues within the brief time window of masked priming. In other words, in the present study, English (L2) primes, written in an alphabetic script with relatively transparent orthography-to-phonology mappings, may allow for rapid and robust phonological activation, which can then spread to Korean (L3) targets and facilitate processing. In contrast, Lim and Ahn (2024) used Korean monosyllabic forms as primes, which, despite the relatively transparent orthography-to-phonology mapping of Korean, have been shown to be processed less efficiently than multisyllabic words in Korean word recognition (Oh et al., 2007), potentially providing relatively limited phonological information during early processing. As a result, phonological activation from Korean (L3) primes may be weaker or slower, reducing the likelihood of effective cross-linguistic activation. This interpretation is further supported by previous findings showing a lack of phonological priming from Korean (L1) to English (L2) when monosyllabic Korean homophone primes were used, despite Korean being the participants’ L1 (Kim and Davis, 2003; Lim and Christianson, 2023). This pattern is unlikely to be solely attributable to differences in lexical familiarity, given that the stimuli were adapted from previous studies using relatively high-frequency words (Kim and Davis, 2003; Lim and Christianson, 2023).

Thus, this account highlights the importance of the accessibility and processing efficiency of phonological information during online lexical access, in addition to the existence of shared phonological representations across languages. Although Li et al. (2024) discussed the relative speed of phonological processing in terms of script differences (logographic vs. alphabetic), the present findings together with their observations further imply that the strength of phonological priming may be influenced by the early availability of phonological information during word recognition. This interpretation, in turn, extends Li et al.’s processing-speed explanation beyond script type, suggesting that, even in non-logographic languages, phonological priming can be influenced by the ease with which phonological information can be activated from the prime. However, this account remains speculative and requires further empirical investigation.

### 5.2 The role of proficiency in phonological activation

Another important finding of the present study concerns the role of language proficiency in lexical decision performance. Crucially, the results revealed a dissociation between L2 and L3 proficiency effects. Specifically, higher English (L2) proficiency was associated with slower response times, whereas higher Korean (L3) proficiency was associated with faster response times in Korean (L3) lexical decision. Notably, the inhibitory effect of L2 proficiency was contrary to the original prediction, given that higher language proficiency has generally been associated with more efficient lexical processing and faster word recognition in previous research (Veivo and Järvikivi, 2013; Rayner, 2009). In addition, neither L2 nor L3 proficiency interacted with prime condition, indicating that proficiency did not influence the magnitude of the phonological priming effect itself. Rather, proficiency influenced overall lexical decision performance independently of the priming effect. This pattern suggests that proficiency does not uniformly facilitate processing but instead modulates lexical decision performance in different ways depending on the status of the language involved.

The facilitative effect of Korean proficiency suggests that higher L3 proficiency enhances the efficiency of lexical access in L3 as predicted, leading to more rapid and automatic word recognition. What is more intriguing, however, is the finding that higher L2 proficiency was associated with slower response times in Korean lexical decision, as this does not conform to the commonly observed facilitative effects of proficiency in bilingual and multilingual processing. In many previous studies, higher proficiency has typically been associated with faster and more efficient lexical access (Veivo and Järvikivi, 2013; Rayner, 2009). In the present study, however, increased L2 proficiency appears to occur greater processing costs, resulting in slower response times as L2 proficiency increases (see Figure 1). One possible explanation concerns the nature of lexical decision itself as pointed out by Ellias et al. (2025). In lexical decision tasks, responses are not driven by a single type of information, but can rely on multiple levels of representation, including orthographic form, phonological structure, and semantic information. These different sources of information contribute to the decision process in parallel, and a response is made once enough consistent evidence has accumulated. In this context, higher L2 proficiency may lead to stronger and more automatic activation of English lexical representations across multiple linguistic levels. Since the English primes shared only phonological overlap with the Korean targets, however, this broader activation may have introduced competing information during L3 lexical decision, thereby increasing processing demands. In this sense, stronger activation from the non-target language may interfere with target-language processing. The present interpretation is partly supported by Chen (2026), who found that although grammatical interference decreased with increasing proficiency, lexical interference remained particularly salient at higher proficiency levels, suggesting that lexical-level cross-linguistic competition persists more in advanced learners.

The present findings become particularly informative when considered alongside Lim and Ahn (2024). Whereas higher English (L2) proficiency was associated with slower responses in the present L2-to-L3 priming study, higher Korean (L3) proficiency was associated with faster responses in their L3-to-L2 priming study. Although the mechanisms underlying this contrast remain unclear, the findings suggest that proficiency may influence lexical processing differently depending on the status and direction of the languages involved. At present, however, the available evidence remains limited, and further empirical research will be necessary to determine whether this apparent asymmetry reflects a robust characteristic of trilingual lexical processing or a pattern specific to particular language combinations and task contexts.

Further insight into the role of proficiency comes from Lim and Christianson (2023), which examined phonological priming from English (L2) to Korean (L1) in Korean-English bilinguals but found no significant effect of L2 proficiency. One key difference between that study and the present one is the status of the target language. In Lim and Christianson (2023), the target language was Korean as an L1, which is typically more stable, dominant, and less susceptible to cross-linguistic interference from a non-native language. As a result, lexical decision in L1 may not have been substantially affected by variation in L2 proficiency. In contrast, in the present study, Korean, the target language, functioned as an L3, a less established language for the participants. This difference in target-language status may have made L3 lexical processing more susceptible to interference from L2, thereby allowing L2 proficiency to modulate response times. In other words, the present findings raise the possibility that proficiency effects may emerge more readily when the target language is relatively weak or less dominant and therefore more susceptible to influence from another language.

Overall, these findings suggest that proficiency effects in trilingual phonological priming may be influenced by factors such as language direction, relative language strength, representational stability, and the balance between facilitation and competition. From this perspective, proficiency should not be treated merely as a background variable that always improves processing efficiency. Rather, proficiency should be considered an important factor in explaining the dynamics of cross-linguistic activation, sometimes facilitating lexical access and sometimes increasing interference, depending on how activation is distributed within the trilingual lexical system.

Despite the theoretical plausibility of our explanation for the observed proficiency effects, the present interpretation should still be treated with considerable caution. Our findings are consistent with the possibility that cross-linguistic activation and competition contribute to lexical decision performance. However, further evidence from methodologies that can more directly track the time course of activation and competition, such as eye-tracking and neurocognitive measures, will be necessary to more precisely characterize the underlying mechanisms responsible for these proficiency effects. In addition, because the present findings are based on a specific language combination and task context, it remains unclear to what extent these proficiency-related dynamics generalize across different trilingual populations, modalities, and experimental paradigms. Much more behavioral evidence will therefore be necessary before our interpretations of these findings can be more confidently generalized across multilingual populations and task contexts.

### 5.3 Implications, limitations, and future directions

Taken together, the present study has important implications for theoretical models of multilingual lexical processing. First, the observed cross-linguistic phonological activation is consistent with the general assumption of non-selective lexical access proposed in models such as the BIA+ model (Dijkstra and van Heuven, 2002), which has been extensively supported in bilingual research. The present findings extend this line of research by demonstrating that phonological similarity alone can give rise to cross-linguistic activation between L2 and L3 in trilingual speakers, even in the absence of semantic overlap. This suggests that language-nonselective phonological activation is not restricted to the conventional L1–L2 relationship examined in bilingual research but can also be observed between two non-native languages in L3 lexical processing. The discussion based on the comparison with Lim and Ahn (2024) highlights the need for models of multilingual lexical processing to move beyond uniform assumptions of cross-linguistic activation. The directional asymmetry and proficiency-related effects observed across the two studies suggest that cross-linguistic activation is not fixed, but may vary depending on processing direction and the relative status and strength of the languages involved. Thus, these findings suggest that models of multilingual lexical processing may need to account for how cross-linguistic activation is shaped by the relative strength of the prime and target language representations, language proficiency, and the linguistic properties of the languages involved. Despite its contributions, the present study has several limitations that should be considered when interpreting the findings. First, the study focused on a specific language combination involving Chinese, English, and Korean, a limitation that is common in behavioral studies of this kind. While this combination provides a useful window into cross-linguistic phonological activation with different scripts and phonological structures, it remains unclear whether the observed patterns generalize to other language combinations. Future research should examine whether similar asymmetries and proficiency effects generalize across different language combinations and speaker profiles.

Another important direction for future research concerns modality. The present study focused on visual word recognition, in which phonological activation is derived from orthographic input. It remains an open question whether similar patterns would be observed in auditory word recognition, where phonological information is directly available. Investigating phonological priming in auditory tasks could help clarify whether the asymmetries observed in the present study are specific to orthographically mediated processing or reflect more general properties of phonological representation in multilingual speakers.

Finally, future research could further investigate how task demands modulate phonological activation in trilingual lexical processing. Different tasks, such as lexical decision, naming, semantic judgment, or translation, impose different demands on the activation and selection of lexical representations, as shown in previous studies demonstrating that task differences can lead to distinct patterns of cross-linguistic activation (Kim and Davis, 2003; Ellias et al., 2025). Examining whether the present patterns of phonological priming and proficiency-related modulation generalize across tasks would provide further insight into the mechanisms underlying multilingual lexical processing.

## 6 Conclusion

The present study demonstrates that phonological similarity alone is sufficient to drive cross-linguistic activation across non-native languages in trilingual lexical processing. By employing homophone pairs that lack semantic and orthographic overlap, the present study examined phonological priming more directly than previous work, which has largely relied on cognates or translation equivalents. In doing so, it provides new evidence that phonological similarity alone can trigger cross-linguistic activation in trilingual lexical access, suggesting shared phonological representations between non-native languages. In addition, by treating proficiency as a continuous factor, the study highlights its critical role in shaping lexical access, showing that proficiency may shape activation dynamics across languages in distinct ways rather than serving as a general facilitatory factor. When considered alongside Lim and Ahn (2024), the present findings further suggest a directional asymmetry in phonological priming, with stronger effects observed in the L2-to-L3 direction than in the reverse direction. This pattern indicates that the extent of phonological priming depends on the strength of the linguistic representations and the speed with which phonological information becomes available during processing. These findings suggest that cross-linguistic phonological activation is not only non-selective, but also dynamically influenced by language direction, representational strength, and proficiency.

Although the present findings provide important insights into phonological processing in trilingual lexical access, they are based on a specific language combination and task context. Much more empirical research will be necessary to determine the extent to which the present findings generalize across different language combinations, proficiency profiles, modalities, and task demands. Such work will contribute to a more comprehensive understanding of how phonological information supports cross-linguistic activation in trilingual lexical processing.

## Data Availability

The raw data supporting the conclusions of this article will be made available by the authors, without undue reservation.
