# Supplementary material for: Cross-linguistic phonological activation in Chinese– English–Korean trilinguals
Source: Front Psychol. 2026 Sep 22;17:1894710. doi: 10.3389/fpsyg.2026.1894710 (PMC13638598; doi:10.3389/fpsyg.2026.1894710)
Supplement: Supplementary file 1 [file Supplementary_file_1.docx]

Appendix A. Experimental Stimuli

| **Target** | **Related prime** | **Unrelate prime** |
| --- | --- | --- |
| 비 | bee | son |
| 돌 | doll | raid |
| 문 | moon | bid |
| 위 | we | soul |
| 영 | young | week |
| 책 | check | race |
| 북 | book | step |
| 불 | bull | yard |
| 피 | pea | sand |
| 빛 | bit | drip |
| 칠 | chill | way |
| 풀 | pull | quit |
| 밭 | but | rank |
| 밀 | mill | blue |
| 인 | inn | sake |
| 밑 | meet | sail |
| 힘 | him | ten |
| 붓 | boot | test |
| 낮 | not | wait |
| 티 | tea | work |
| 일 | ill | your |
| 눈 | noon | air |
| 겉 | gut | best |
| 키 | key | fall |
| 집 | jeep | lamb |
| 볼 | bowl | full |
| 토 | toe | hard |
| 넷 | net | fear |
| 암 | arm | time |
| 숲 | soup | corn |
